# Internet Use for Searching Information on Medicines and Disease: A Community Pharmacy–Based Survey Among Adult Pharmacy Customers

**DOI:** 10.2196/ijmr.5231

**Published:** 2016-07-13

**Authors:** Simona Lombardo, Marco Cosentino

**Affiliations:** ^1^ Center for Research in Medical Pharmacology University of Insubria Varese Italy

**Keywords:** Internet, health information, information searching behavior, medicines, diseases, survey

## Abstract

**Background:**

The Internet is increasingly used as a source of health-related information, and a vast majority of Internet users are performing health-related searches in the United States and Europe, with wide differences among countries. Health information searching behavior on the Internet is affected by multiple factors, including demographics, socioeconomic factors, education, employment, attitudes toward the Internet, and health conditions, and their knowledge may help to promote a safer use of the Internet. Limited information however exists so far about Internet use to search for medical information in Italy.

**Objective:**

The objective of this study was to investigate the use of the Internet for searching for information on medicines and disease in adult subjects in Northern Italy.

**Methods:**

Survey in randomly selected community pharmacies, using a self-administered questionnaire, with open and multiple choices questions, was conducted.

**Results:**

A total of 1008 participants were enrolled (59.5% women; median age: 43 years; range: 14-88 years). Previous use of the Internet to search for information about medicines or dietary supplements was reported by 26.0% of respondents, more commonly by women (30.00% vs 20.10% men, *P*<.001), unmarried subjects (32.9% vs 17.4% widowed subjects, *P*=.022), and employed people (29.1% vs 10.4% retired people, *P*=.002). Use was highest in the age range of 26 to 35 (40.0% users vs 19.6% and 12.3% in the age range ≤25 and ≥56, respectively, *P*<.001) and increased with years of education (from 5.3% with 5 years, up to 41.0% with a university degree, *P*<.001). Previous use of the Internet to search for information about disease was reported by 59.1% of respondents, more commonly by women (64.5% vs 51.0% males, *P*<.001), unmarried subjects (64.2% vs 58.5% married or divorced subjects and 30.4% widowed subjects, *P*=.012), unemployed people (66.7% vs 64.0% workers and 29.9% retired people, *P*<.001). Use was highest in the age range of 26 to 35 (70.1% vs 64.4% in both 36-45 and 46-55 ranges and 35.1% in ≥56, *P*<.001) and increased with years of education (from 12.5% with 5 years up to 66.7% with 13 years and 68.6% with a university degree, *P*<.001). Retrieved information was rated as satisfactory by about 87.5% (88.1% women and 86.2% men, *P*=.562). Recent use of medicines or dietary supplements was associated with more frequent use of the Internet to search for disease and drugs.

**Conclusions:**

The study provides detailed information on the use of the Internet for searching for information on medicines and disease in the Italian population. Gender, age, social status and level of education, and the previous use of medicines, affect searching behaviors and use patterns. Results can support educational interventions to promote the retrieval of high-quality information by Internet users and health professionals advising patients about appropriate use of Internet for health-related purposes.

## Introduction

The Internet is currently a major source of health- and medical-related information. People using the Internet for health-related searches are estimated to be up to more than 70% of Internet users in both the United States [1,2] and Europe [3]. In particular, the European survey, including 7 countries (Norway, Denmark, Germany, Greece, Poland, Portugal, and Latvia) and a total of 7934 respondents reported percentages of Internet users searching for health-related information in the range of 30% to 62% of the total sample (and 54%-79% of total Internet users) with wide differences among countries [3].

Specific diseases or health-related problems are acknowledged as the main determinants in deciding to search for medical information on the Internet; however, health information searching behavior on the Internet is affected by multiple factors, including demographics, socioeconomic factors, education, employment, attitudes toward the Internet, and health conditions [1-3]. Knowledge of such factors may allow the promotion of a safer use of the Internet for health purposes, also in consideration of potential dangers such as the dissemination of inaccurate information and the inappropriate use [4-6]. The Internet has indeed an enormous potential for health promotion, which however requires the development of a critical usership and the collaboration of health care professionals [7]. To meet such requirements, detailed knowledge of Internet contents (eg, [8,9]) and of the attitudes of their users is mandatory.

The use of the Internet to search for medical information in Italy has received so far limited attention, in a few specific populations, such as amyotrophic lateral sclerosis patients and caregivers [10], and pregnant women [11,12], and concerning specific issues, such as chronic obstructive pulmonary disease patients' knowledge regarding cardiopulmonary resuscitation [13]. Only 1 survey currently exists addressing the extent of Internet use to retrieve medical information in a sample of adults selected among parents of public school students in Southern Italy [14].

### Objectives

The objectives of this study were to investigate the use of the Internet for searching for information on medicines and disease in a sample of adult subjects recruited among pharmacy customers in Northern Italy, with particular regard to personal use of medicines and dietary supplements and individual attitudes toward the use of the Internet for the purchase of goods, to provide evidence about the association between Internet use and specific factors (eg, age, gender, employment, and so forth). Italian pharmacies have a monopoly on prescription medications. In addition, they sell over-the-counter medications and nonmedical products such as cosmetics, medical devices, dietary supplements, special foods (for people with diabetes, coeliac disease, and so forth). Italian pharmacies do not deal with many nonmedical items (eg, beverages, food, magazines, wrapping paper and household items, and so forth). Pharmacy customers in Italy are therefore highly likely to have specific medical or health problems regarding themselves, their relatives, and so forth.

## Methods

### Participants

This survey was carried out from October 2010 till July 2011 in 5 randomly selected community pharmacies in the province of Como, in Northern Italy. Participants were pharmacy customers who were approached consecutively and invited to participate in the study when fulfilling the following inclusion criteria: being aged ≥18 years, giving written informed consent.

### Questionnaire

Persons who agreed to participate filled out a self-administered questionnaire (Multimedia Appendix 1), with open and multiple choices questions, structured into 3 sections devoted to the collection of the following data:

1. Sociodemographic: age, gender, level of education, employment, and marital status.

2. Use of medicines and dietary supplements in the previous 6 months.

3. Internet use: searching of information about disease, medicines, dietary supplements, attitudes toward Internet purchase of goods in general, of medicines, and of dietary supplements.

### Analysis

Collected data were recorded into a digital archive (Microsoft Excel). Drugs were classified according to the World Health Organization (WHO) Anatomical Therapeutic Chemical Classification System, whereas diseases were classified according to the WHO International Statistical Classification of Diseases, Injuries and Causes of Death, 10th Edition. Records were validated according to the International Quality Standard ISO 2859 guidelines (ISO 2859-4:2002), and the database was considered suitable for analysis. Before the analysis, each record was checked for intrasection and intersection coherence. Statistical approach was based on distribution of responses. Gaussian distribution was checked by means of the D'Agostino and Pearson omnibus normality test. Proportions were compared using chi-square analysis, and means were compared using Student's *t*-tests. Odds ratios and corresponding 95% CIs were obtained using the Woolf logit method. Analyses were performed using a commercial software (GraphPad Prism version 5.00 for Windows; GraphPad Software, San Diego, CA, USA).

## Results

### Participants

The survey enrolled a total of 1008 participants. Their demographic details are shown in Table 1. The median age was 43 years (interquartile range: 34-51), with a range of 14 to 88 years. Most of the participants were married or cohabiting (61.8%) or single (30.3%), with no differences between women and men. Nearly 70% of the participants had secondary school or university degrees. University degrees were declared more frequently by women than men (26.0% vs 19.4%). Nearly 73% of respondents were employed, and 10% were retired. In comparison to women, men were more likely to be employed (79.5% vs 68.1%) or retired (13.7% vs 7.5%), whereas women were more likely to be unemployed (2.6% vs 0.7%) and housewives (15.2% vs 0.0%).

**Table 1:** Demographics of participants in the survey.

Item		Total (%)	Female (%)	Male (%)	*P* ^a^
		1008 (100)	600 (59.5)	408 (40.5)	
Age (years)^b^					
	Mean standard deviation	43.3 13.9	42.6 13.8	43.8 14.0	.999
Age distribution					.169
	≤25	105 (10.6)	66 (11.1)	39 (9.8)	
	26-35	168 (17.0)	109 (18.4)	59 (14.9)	
	36-45	299 (30.2)	186 (31.4)	113 (28.5)	
	46-55	249 (25.2)	142 (23.9)	107 (27.0)	
	≥56	168 (17.0)	90 (15.2)	78 (19.7)	
Marital status^c^					.100
	Single	296 (30.3)	183 (31.5)	134 (33.8)		
	Married or cohabiting	603 (61.8)	356 (61.4)	247 (62.4)	
	Separated or divorced	54 (5.5)	22 (3.8)	11 (2.8)	
	Widowed	23 (2.4)	19 (3.3)	4 (1.0)	
Education (years)^d^					.004
	5	41 (4.3)	31 (5.5)	10 (2.5)	
	8	249 (26.0)	131 (23.3)	118 (29.7)	
	13	445 (46.4)	253 (45.1)	192 (48.4)	
	>13	223 (23.3)	146 (26.0)	77 (19.4)	
Employment^e^					<.001
	Yes	717 (72.7)	399 (68.1)	318 (79.5)	
	No	18 (1.8)	15 (2.6)	3 (0.7)	
	Retired	99 (10.0)	44 (7.5)	55 (13.7)	
	Student	63 (6.4)	39 (6.7)	24 (6.0)	
	Housewife	89 (9.0)	89 (15.2)	0 (0.0)	

^a^Women versus men.

^b^Missing answers: 19.

^c^Missing answers: 32.

^d^Five years correspond to completion of primary school, 8 to secondary school, 13 to high school, more than 13 indicate enrollment into a university course. Missing answers: 50.

^e^Missing answers: 22.

### Information on Medicines

Overall, of 991 respondents, 258 (26.0%) reported previous use of the Internet to search for information about medicines or dietary supplements. Use of the Internet for this purpose was much more common among women in comparison to men (30.0% vs 20.1%, *P*<.001), and it was highest in the age range of 26-35 and lowest in the age range of ≤25 and ≥56 (40.0% users vs 19.6% and 12.3%, respectively, *P*<.001). Higher use was reported by unmarried subjects in comparison to widowed subjects (32.9% and 17.4%, respectively, *P*=.022) and by people with an employment in comparison to retired people (29.1% and 10.4%, respectively, *P*=.002), and use increased with years of education (from 5.3% with 5 years up to 41.0% with a university degree, *P*<.001).

Respondents most frequently searched for information on the following medications: central nervous system (CNS) drugs (22.3%), gastrointestinal drugs (17.9%), musculoskeletal drugs (15.2%), antibiotics (10.7%), genitourinary system drugs, and sex hormones (9.8%), antitumor drugs (6.2%), hormones and drugs for the respiratory system (5.4% each).

Among dietary supplements, information most frequently searched was about products containing minerals, vitamins, amino acids or proteins (20.9%), products for sport activities (13.4%), for menopause (9.0%), for body weight (7.5%), and cholesterol and for digestive tract (6.0% each).

Retrieved information was rated as satisfactory by 89.2% among both women and men.

Of 999 respondents, 684 (68.5%) were aware of the possibility to purchase medicines on the Internet (65.0% women vs 73.5% men, *P*=.005), and awareness increased with age (from 41.7% at ≤25 up to 76.6% at 46-55, *P*<.001). Awareness was higher in married or divorced subjects in comparison to widowed subjects (73.3%, 72.2%, and 47.8%, respectively, *P*<.001) and in people with an employment in comparison to people without an employment and to students (71.3%, 55.6%, and 55.6%, respectively, *P*=.026), and awareness increased with years of education (from 45.0% with 5 years up to 76.2% with 13 years or more, *P*<.001).

Only 9.2% of respondents had a positive opinion about purchase of medicines on the Internet (6.1% women vs 15.9% men, *P*=.001).

Possibility to purchase dietary supplements on the Internet was known by 70.3% of respondents (67.7% women vs 74.0% men, *P*=.039), and awareness increased with age (from 50.5% at ≤25 up to 75.9% at 46-55, *P*<.001), and it was rated as positive by only 13.2% (12.2% women vs 14.7% men, *P*=.430). Awareness was higher in married or divorced subjects in comparison to widowed subjects (72.3%, 75.0%, and 39.1%, respectively, *P*=.005) and in people with an employment in comparison to people without an employment (73.6% and 55.6%, respectively, *P*=.024), and awareness increased with years of education (from 42.5% with 5 years up to 81.9% with 13 years or more, *P*<.001).

### Information on Disease

Previous use of the Internet to search for information about disease was reported by 590 of 999 respondents (59.1%). More women than men used the Internet for this purpose (64.5% vs 51.0%, *P*<.001). Use of the Internet to search for information about disease was highest in the age range of 26-35 (70.1%) and 36-45 and 46-55 (both, 64.4%) and lowest in the age range of ≥56 (35.1%, *P*<.001). Highest use was reported by unmarried subjects (64.2%), followed by married or divorced subjects (58.5%), and widowed subjects declared the lowest use (30.4%, *P*=.012). Higher use was reported by people without an employment (66.7%), followed by workers (64.0%), and lower use by retired people (29.9%, *P*<.001). Use increased with years of education (from 12.5% with 5 years up to 66.7% with 13 years and 68.6% with a university degree, *P*<.001).

Most of the respondents (32.9%) declared that they sought information on several unspecified disease. Specified diseases most commonly sought were: cancer (19.0%), CNS disease (11.6%), infectious disease (10.5%), musculoskeletal disease (10.1%), gastrointestinal disease (9.5%), endocrine and metabolic disease (8.3%), cardiovascular disease (7.7%), genitourinary system disease (6.6%), skin disease (6.4%), traumatic disease and intoxication (4.3%), respiratory disease (4.1%), mental and behavioral disturbances (2.7%), abnormal laboratory results (1.5%), hematopoietic disease (1.2%), and ocular disease, ear disease, pregnancy and partum, malformations (all less than 1.0%).

Retrieved information was rated as satisfactory by about 87.5% (88.1% women and 86.2 men, *P*=.562).

### Relationship With Previous Use of Medicines or Dietary Supplements

Respondents who reported use of medicines or dietary supplements in the previous 6 months made more frequent use of the Internet to search for both disease and drugs (Table 2).

**Table 2:** Relationship between Internet use for information on medicines and disease and previous use of medicines or dietary supplements.

Use in the last 6 months	Looked for information on Internet							
	**Disease**		Odds Ratio (95% CI)	*P*	**Medicines/** **dietary supplements**		Odds Ratio (95% CI)	*P*
	Yes, n (%)	No, n (%)			Yes, n (%)	No, n (%)		
**Medicines**								
Yes (n=788)	477 (60.5)	304 (38.6)	1.40 (1.02-1.92)	.041	218 (27.7)	560 (71.1)	1.70 (1.14-2.54)	.009
No (n=195)	102 (52.3)	91 (46.7)			35 (17.9)	153 (78.5)		
								
**Dietary supplements**								
Yes (n=788)	249 (65.5)	130 (34.2)	1.57 (1.20-2.05)	<.001	133 (35.0)	243 (63.9)	2.17 (1.62-2.90)	<.001
No (n=195)	330 (54.3)	271 (44.6)			120 (19.7)	476 (78.3)		

## Discussion

### Principal Findings

The results of the present survey, involving more than 1000 participants in Northern Italy, show that the use of the Internet to search for information on medicines and disease is widespread, however, to a different extent. Nearly 60% of respondents used the Internet to search for information about disease, while previous use of the Internet to search for information on medicines or dietary supplements was reported by only 26% of respondents. The only survey so far available in the Italian population was published in 2013 and reported about 53% adult people using the Internet to retrieve information about drugs and between 70% and 86% about disease diagnosis and treatment [14]. Such survey was conducted among parents of public school students, whereas our survey recruited pharmacy customers. As a consequence, the former might have overestimated the frequency of healthy subjects while excluding elderly people, whereas the present survey might have included more people with health problems. Both of them therefore might not precisely estimate Internet information searching in general among all adults. Further implications of the sampling strategy are discussed below (see “Limitations”).

Despite the aforementioned huge difference between Internet searching for disease and drugs, the profile of information searchers was similar: searching for health-related information on the Internet was more common among women in comparison to men, in the age range of 26-35, among unmarried subjects and employed people and increased with years of education, being highest in those with a university degree. In addition, use of medicines or dietary supplements in the previous 6 months increased the odds of Internet use to search for both disease and drugs. On the contrary, the use of the Internet for such purposes was lower in men, in the age range of ≤25 and ≥56, as well as among widowed subjects or retired people, and in people with only a few years of education. The profile of information searchers is in agreement with those of previous reports showing that characteristics associated with Internet use for health-related information included being younger, women, having a higher level of education, and suffering for chronic conditions [14-16]. Indeed, in our study, use of medicinal products in the previous 6 months can be considered as a proxy for both acute and chronic health problems.

Most searched diseases were cancer and CNS disease, followed by infectious disease, musculoskeletal disease, and gastrointestinal disease. Among searched medicines, CNS drugs ranked first; however, antitumor drugs ranked only seventh, after gastrointestinal drugs, musculoskeletal drugs, antibiotics, genitourinary system drugs, and sex hormones, suggesting the lack of any direct correlations between the perceived need for information on disease and medicines. It is likely that different personal factors play a role in deciding to search for information about medicines and about disease, and this issue might deserve further attention in future studies.

Dietary supplements most frequently sought were: minerals, vitamins, amino acids, and products for sport activities in general, followed by products for menopause and body weight. This is in agreement with recent marketing data, showing that in Italy, dietary supplements are increasingly used not only for disease prevention but also for performance enhancement, and that use of dietary supplements for disease prevention is more common about elderly subjects and in particular among women [17].

According to our results, about 70% of the respondents were aware of the possibility to purchase medicines or dietary supplements on the Internet. Interestingly, people aware of this possibility were mainly men, and awareness increased with age and with years of education. Although education is a well-known strong predictor of access to the Internet [18], and in this survey, it was associated, together with female gender and younger age, also with the use of the Internet for searching for information about disease and medicines, male gender and age seem to be specific for the knowledge about the use of the Internet for purchasing purposes. Indeed, different attitudes and perceptions have been reported for women and men with regard to purchasing goods and services on the Internet [19]. Anyway, it must be emphasized that, at the time of this survey, in Italy, it was not possible to purchase medicines on the Internet, and this could also well explain the usually negative opinion expressed by more than 90% of the respondents about purchase of medicines on the Internet. Remarkably, in this regard, women were even more negative than men.

Italy was not among the European countries surveyed in the study by Andreassen et al [3]. In the study, factors positively associated with the use of the Internet for health purposes were young age, female gender, higher education, white-collar or no paid job, visits to the general practitioner during the past year, long-term illness or disabilities, and a subjectively perceived good health [3]. In our survey, factors affecting Internet use for health purposes were female gender, unmarried condition, employed people, age range of 26-35, and higher education. We did not investigate factors such as visits to the general practitioner, presence of illness or disabilities, or subjective perception of one's own health. Comparison of results shows however that although some factors may exert similar roles (eg, female gender, higher education), others may differ depending on the specific context, further supporting the need for focused research.

### Limitations

The main limitation of this study is that it was performed in Northern Italy, and therefore, it might not represent all the Italian population. Furthermore, the sample was recruited among pharmacy customers, potentially leading to a selection bias toward people preferentially purchasing medicines through conventional means. The surveyed population, compared with the general population of Italy [20], probably oversampled women (59% vs 51%), undersampled people aged ≤35 years (28% vs 36%) and ≥56 (17% vs 33%), and oversampled people aged 36-55 years (55% vs 31%). Moreover, in our sample, the proportion between married or cohabiting people and single people was 2:1, whereas in the general population, it is nearly 1:1 [20]. Such differences must be taken into account.

### Conclusions

The present investigation provides detailed information on the use of the Internet for searching for information on medicines and disease in the Italian population. The Internet is among the main sources of health- and medical-related information, with an increasing number of Internet users searching for health-related information in the absence of any medical or expert supervision or advice. It is therefore of paramount importance to assess information searching behaviors and patterns, as well as the relevant associated factors, to allow the promotion of a safer use of the Internet for health purposes. Our study provides evidence about the role of gender, age, social status, and level of education, together with details on the health-related topics most commonly sought and their relationship with the previous use of medicines. Such results can be used to support educational interventions aimed at improving the ability of Internet users to select and preferentially retrieve high-quality information, as well as the ability of health professionals to assist and advise their patients about the appropriate use of Internet for health-related purposes.

## Appendix

Multimedia Appendix 1

## Abbreviations

CNS: central nervous system
WHO: World Health Organization
