# Supplementary material for: Internet Use for Searching Information on Medicines and Disease: A Community Pharmacy–Based Survey Among Adult Pharmacy Customers
Source: Interact J Med Res. 2016 Jul 13;5(3):e22. doi: 10.2196/ijmr.5231 (PMC4988641; doi:10.2196/ijmr.5231)
Supplement: Multimedia Appendix 1 [file ijmr_v5i3e22_app1.pdf]

FARMACI E INTERNET:
INDAGINE SU MEDICINALI, INTEGRATORI ALIMENTARI E WEB

Parte I – Dati personali

1.1 Sesso: ☐ M ☐ F Età: ____

1.2 Stato civile: ______________

1.3 Ha figli? ☐ Si ☐ No

1.4 Qual è il suo titolo di studio? ____________________________________

1.5 Qual è la sua occupazione? ______________________________________

1.6 Qual è il suo luogo di residenza? _______________________________

Parte II – Uso di farmaci e di integratori alimentari

2.1 Negli ultimi sei mesi ha utilizzato:
FARMACI? ☐ Si ☐ No
INTEGRATORI? ☐ Si ☐ No

Parte III – Internet

3.1 Ha mai comprato in Internet?
☐ Si ☐ No

3.2 Ha mai cercato informazioni su una malattia in Internet?
☐ Si ☐ No

Se si, per quale/i? ____
Se si, è rimasto/a soddisfatto/a? ____

3.3 Ha mai cercato informazioni su un farmaco e/o un integratore alimentare in Internet?
☐ Si ☐ No

Se si, per quale/i? ____
Se si, è rimasto/a soddisfatto/a ? _______________________________________

3.4 E' a conoscenza della vendita dei farmaci in Internet?
☐ Si ☐ No

Che cosa ne pensa? ____
____

3.5 E' a conoscenza della vendita degli integratori alimentari in Internet?
☐ Si ☐ No

Che cosa ne pensa? ____
____

Progetto di ricerca: **FARMACI E INTERNET: INDAGINE SU MEDICINALI, INTEGRATORI ALIMENTARI E WEB**

Gent.ma Signora, Egr. Signore:

La invitiamo a partecipare a un progetto che intende investigare sui fattori che inducono all'acquisto di un farmaco e/o un integratore alimentare.

Il progetto è coordinato dalla Dr.ssa Simona Lombardo, la quale sta frequentando il Corso di Dottorato di Ricerca in Farmacologia Sperimentale e Clinica presso il Dipartimento di Medicina Clinica dell' Università degli Studi dell' Insubria di Varese.

La partecipazione al progetto consisterà nella compilazione del questionario allegato a questa lettera. Il questionario è anonimo e in nessun modo le risposte fornite potranno essere ricondotte alla sua persona.

La ringraziamo anticipatamente per la sua preziosa collaborazione.

Distinti saluti

La responsabile del progetto
Dr.ssa Simona Lombardo

Il docente-guida
Prof. Marco Cosentino

Per informazioni e chiarimenti:
Dr.ssa Simona Lombardo
Dipartimento di Medicina Clinica
Sezione di Farmacologia Sperimentale e Clinica
Università degli Studi dell'Insubria
Via Ottorino Rossi n. 9
21100 Varese VA
Tel. (segreteria): +39 0332 217401/397401
Fax: +39 0332 217409/397409
E-mail: simona.lombardo@uninsubria.it

Consenso alla partecipazione e al trattamento dei dati personali.

Il/la sottoscritto/a (nome e cognome) ___________________________________ esprime il proprio consenso alla partecipazione al progetto nonché al trattamento dei propri dati nel pieno rispetto del D.L.vo n. 196/2003 e norme successive.

Luogo e data _______________ Firma _________________
